# Tailored resection of spinal dumbbell schwannomas using transforaminal and modified epidural trans dural sleeve approaches with hemilaminectomy

**DOI:** 10.3389/fsurg.2025.1684406

**Published:** 2025-11-13

**Authors:** Qiaowei Wu, Yihua Rao, Xiaohai Zhang, Yulong Shen, Dongxu Wu, Zhicheng Shu, Kebo Liu, Kun Luo, Jing Yao, Jianbiao Liu, Weifeng Li, Changyong Yang, Jianping Wen

**Affiliations:** 1 Department of Neurosurgery, Hunan University of Medicine General Hospital, Huaihua, China; 2 Department of Neurosurgery, Huaihua Central Hospital, Huaihua, China

**Keywords:** schwannomas, dumbbell-shaped, epidural approach, hemilaminectomy, laminectomy

## Abstract

**Background and purpose:**

Surgical resection of dumbbell-shaped spinal schwannomas, which involve both intra-extradural and intra-extraspinal components, presents significant technical challenges. This study aimed to evaluate the safety and efficacy of the transforaminal and modified epidural trans dural sleeve approaches with hemilaminectomy without dural incision for the tailored resection of spinal dumbbell-shaped tumors in comparison with conventional laminectomy.

**Methods:**

This was a retrospective, observational cohort study. Between May 2017 and November 2024. A total of 44 patients with extra-intraspinal dumbbell-shaped tumors who underwent tumor resection were included in the analysis. Procedure-related data, postprocedural serious adverse events (SAEs), clinical and imaging outcomes were evaluated.

**Results:**

All patients (44 tumors) were successfully treated, of which 20 patients were treated through the epidural trans-dural sleeve approach (epidural approach group) and 24 patients were treated through the conventional laminectomy and dural incision (conventional approach group). Gross total resection was performed in 95.5% (42/44) patients, with 2 cases of residual tumor in the conventional approach group. A total of 12 (27.3%) patients experienced at least one SAE before discharge, including 3 (15.0%) in the epidural approach group and 9 (37.5%) in the conventional approach group (*P* = 0.095). Blood transfusions were performed in 10 (41.7%) patients in the conventional approach group, and no patients were required in the epidural approach group (*P* = 0.003). Multivariate analysis revealed that intraprocedural estimated blood loss ≥ 350 mL was significantly associated with SAEs during hospitalization (OR: 6.6; 95%CI: 1.5–29.7; *P* = 0.014). During the follow-up duration of 28.0 (9.5, 51.0) months, 42 patients were classified as ASIA grade E, and 2 as grade D. No tumor recurrence was detected during the imaging follow-up.

**Conclusion:**

Epidural trans-dural sleeve approach can be a safe and effective method for treating spinal dumbbell-shaped tumors, with relatively lower intraprocedural blood loss and transfusion rates compared to conventional techniques. Intraprocedural estimated blood loss ≥ 350 mL was significantly associated with SAEs during hospitalization.

## 1 Introduction

Dumbbell-shaped spinal tumors are uncommon growths extending across the dura or the spinal canal. Their constriction at the level of the intervertebral foramen or dural penetration results in the pathognomonic dumbbell morphology (1, 2). Dumbbell-shaped tumors account for approximately 17.5% of all spinal tumors (2, 3). The most common type of spinal dumbbell-shaped tumor is schwannoma, followed by neurofibroma and meningioma (2). Anatomically, dumbbell tumors most frequently occur in the cervical spine, followed by the thoracic and lumbar regions (2, 4). Although most spinal tumors are benign, they typically compress nerve roots and the spinal cord, leading to progressive pain or severe neurological deficits (1–4).

The treatment for spinal dumbbell-shaped schwannomas involves surgical resection of the tumor, which can alleviate clinical symptoms and relieve compression on neural structures. The conventional surgical approach typically involves posterior midline, anterolateral, or posterolateral approaches, and in some cases, a combined anterolateral and posterolateral approach is required to resect spinal dumbbell-shaped tumors (1, 2, 5, 6). Moreover, although the traditional bilateral laminectomy and dural incision for resecting intra-extradural spinal dumbbell-shaped schwannomas provided wide surgical exposure, it was highly invasive and often carried risks of surgery-related spinal cord injuries, spinal instability, and motor or sensory deficits (5–8). To further minimize surgical trauma, hemilaminectomy has gained popularity by resecting only the spinous process root and ipsilateral lamina, thereby reducing postprocedural pain and enhancing spinal stability, making it one of the primary surgical approaches for spinal tumors (9, 10). Some scholars have proposed utilizing the imaging features of tumors and the local anatomical structure of the spine to remove intraspinal dumbbell-shaped tumors through the intervertebral foramen, achieving favorable therapeutic outcomes (11, 12). However, the relatively large surgical incision and dural injury still carry risks of postprocedural spinal cord injury and cerebrospinal fluid leakage (CSF) (7, 13). We attempted to completely resect both the intraspinal and intradural portions of dumbbell-shaped tumors via the intervertebral foramen or hemilaminectomy using an extradural approach along the tumor's natural pathway without dural incision, aiming to minimize spinal cord injury, reduce postprocedural CSF, decrease surgical trauma, and simplify the procedural processes. This retrospective study aimed to investigate the safety and efficacy of the tailored microsurgical resection of intra-extradural spinal dumbbell-shaped schwannomas through the transforaminal and modified epidural trans dural sleeve approaches with hemilaminectomy without dural incision, and compared the outcomes with those of conventional approaches.

## 2 Materials and methods

### 2.1 Study design and patients

This retrospective, observational cohort study assessed the safety and efficacy of the resection of intra-extradural spinal dumbbell-shaped schwannomas via the epidural trans-dural sleeve approach without dural incision (epidural approach group) and conventional bilateral laminectomy with dural incision (conventional approach group) from Hunan University of Medical general hospital and Huaihua central Hospital between May 2017 and November 2024. A total of 44 patients with intra-extradural spinal dumbbell-shaped schwannomas were enrolled in our study.

To be eligible for inclusion in this study, the patients had to meet the following inclusion criteria: (1) patients were 18 years of age or older; (2) tumors were detected by preprocedural magnetic resonance imaging (MRI); (3) the tumor involved the intradural and intraspinal compartments with extension into the spinal foramen, exhibiting a dumbbell-shaped morphology; (4) the tumors were resected via the intervertebral foramen approach, bilateral laminectomy or hemilaminectomy; (5) The pathological results showed that the tumors were schwannomas. Patients were excluded from the study if they met any of the following exclusion criteria: (1) patient had a history of spinal trauma and previous spinal surgery; (2) the patient combined with cerebrovascular diseases or other spinal cord disorders that may affect neurological function assessment; (3) postprocedural imaging and medical records were unavailable/missing.

### 2.2 Data collection

Baseline characteristics, including age, sex, and medical history, were recorded for each patient. The Eden classification categorizes dumbbell-shaped tumors into four types: Type 1 (intra- and extradural), Type 2 (intra-/extradural with paravertebral extension), Type 3 (extradural and paravertebral), and Type 4 (foraminal and paravertebral) (2). The preprocedural and postprocedural motor/sensory functions were evaluated according to the American Spinal Injury Association (ASIA) scale (14). The tumor pathological diagnosis, procedural time, and intraprocedural blood loss were recorded. The preprocedural and postprocedural spinal MRI scans were performed to confirm complete tumor resection. In-hospital serious adverse events (SAEs) were recorded, including postprocedural central nervous system (CNS) infection, CSF leakage, or other threatening events, leading to hospitalizations or prolonged hospitalizations. Clinical function was assessed on admission, post-procedure, at discharge, and during follow-up visits at 3 months, 1 year, and yearly thereafter. If a patient was unable to return to the hospital, outcomes were assessed through a standardized telephone interview. Imaging follow-up with MRI scan was scheduled at 1 year and 2–5 years after the procedure.

### 2.3 Procedure techniques

**Figure 1:**
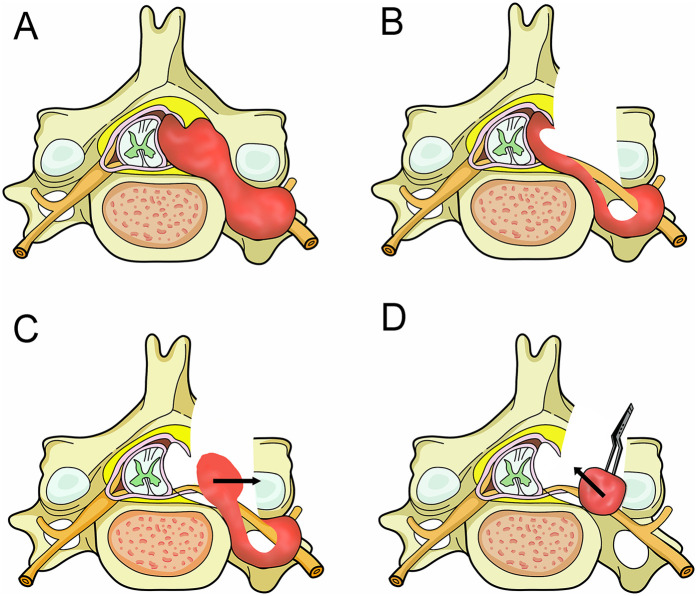
Surgical diagrams illustrating the operation process. **(A)** A dumbbell tumor in the left cervical spine on axial planes, with growth extending into the foramen and compressing the spinal cord. **(B)** After tumor exposure via hemilaminectomy, the tumor in the isthmus was resected first, followed by internal decompression of the mass. **(C)** The intra-canal and subdural portions of the tumor were then gently retracted outward (Arrow), carefully freeing the tumor tissue from surrounding membranous structures. **(D)** Finally, the extraspinal portion of the tumor was removed (arrow).

All surgeries were performed by neurosurgeons with over 25 years of experience in spinal surgery. After satisfactory general anesthesia, the patient was placed in a prone position, and the correct location of the affected vertebra was identified using multi-angle fluoroscopy. For patients undergoing the epidural trans-dural sleeve approach, a posterior midline incision was made, and the skin was incised. The paraspinal muscles were dissected laterally to the root of the transverse process, exposing the ipsilateral hemilamina, facet joint, the extra-canal portion of the tumor, and the intervertebral foramen. For patients undergoing the trans-foraminal approach, if the intervertebral foramen was too narrow to expose and resect the intra-canal portion of the tumor, a micro-drill was used to remove part of the inferior pedicle and facet joint to enlarge the foramen. For patients undergoing hemilaminectomy, an ultrasonic bone cutter and rongeur were used to remove the ipsilateral hemilamina and part of the facet joint while preserving the continuity between the spinous process and the contralateral lamina. The extra-canal portion of the tumor was partially resected first, followed by internal decompression. The intra-canal and subdural portions of the tumor were then gently retracted outward, without retraction of the nerve roots and spinal cord. And then carefully freed the tumor tissue from surrounding membranous structures. Bipolar coagulation was applied to the tumor surface to achieve hemostasis, reduce blood supply, and shrink the tumor volume. This maneuver allowed for the delivery of the intradural tumor component through the expanded neural foramen without the need to incise the adherent dural root sleeve or perform a formal durotomy on the thecal sac. No additional dural incision was made, and the tumor was always retracted gently along its growth direction. The parent nerve root, usually clearly demarcated and located on one side of the tumor, could be carefully preserved during tumor retraction. For tumors extending into the subdural space, the intradural portion was often covered by thin arachnoid membranes but typically had a clear plane of separation. Gentle retraction of the intra-canal portion allowed the tumor to be fully extracted without damaging the arachnoid, preventing cerebrospinal fluid leakage. Finally, the paraspinal muscles and skin were sutured in layers. Patients with minimal bone structure disruption did not require internal fixation and were encouraged to ambulate as soon as possible postoperatively. A schematic diagram of the surgical procedure is shown in Figure 1.

For patients undergoing traditional laminectomy and durotomy, a posterior midline approach was used. The skin was incised, and the paraspinal muscles were dissected to expose the roots of the transverse processes, bilateral laminae, the ipsilateral facet joint, and the extra-canal portion of the tumor. An ultrasonic bone cutter and rongeur were employed to remove the spinous process and bilateral laminae entirely, fully exposing the spinal canal, while partial facetectomy was performed to optimize tumor exposure. Under microscopic guidance, the tumor was resected *en bloc* or piecemeal. For larger tumors, internal decompression was performed using an ultrasonic aspirator before segmental resection. For tumors involving the subdural space, the dura was incised, and the tumor was carefully dissected before dural closure. Patients with significant damage to the spinal bony structures required internal fixation, followed by placement of a surgical drain and layered closure of the paraspinal muscles and skin. In the absence of complications, patients were typically encouraged to ambulate early postoperatively to reduce the risk of deep vein thrombosis.

### 2.4 Statistical analyses

Numerical data conforming to a normal distribution were summarized using means and standard deviations (SDs), whereas skewed variables were reported as medians with interquartile ranges (IQRs). Categorical measures were expressed as frequency counts and proportions. Comparative analyses for continuous variables employed either independent t-tests (for normally distributed data) or nonparametric Mann–Whitney U tests (for non-normal distributions). For categorical comparisons, Fisher's exact test or the chi-square test was applied as appropriate.

To explore the factors influencing SAEs in patients, univariate analysis was first performed on all variables. Variables with a *P*-value < 0.1 in the univariate analysis were then included in the multivariate logistic regression model. In the multivariate analysis, continuous variables were dichotomized into categorical variables based on their optimal cutoff values. All statistical computations were conducted in SPSS (Version 22.0, IBM Corp.), with a two-tailed *P*-value < 0.05 denoting statistical significance.

## 3 Results

### 3.1 Patient and tumor characteristics

Among the 44 patients with intra-extradural dumbbell-shaped tumors enrolled in this study, 20 (45.5%) were in the epidural approach group and 24 (54.5%) in the conventional approach group. The cohort included 27 males (61.4%) and 17 females (38.6%), with a mean age of 50.5 ± 13.3 years. Preprocedural ASIA impairment scale grades were distributed as follows: grade C in 1 patient (2.3%), grade D in 22 patients (50.0%), and grade E in 21 patients (47.7%). Symptom duration prior to procedure ranged from 1 week to 120 months (median: 36 months). Clinical presentations included: pain (81.8%, 36/44), paresthesia (61.4%, 27/44), motor weakness (50.0%, 22/44), and sphincter dysfunction (6.8%, 3/44). Detailed baseline characteristics are presented in Table 1.

**Table 1:** Baseline characteristics.

Variables	EA group (*n* = 20)	CA group (*n* = 24)	*P* value
Male, *n* (%)	14 (70.0)	13 (54.2)	0.283
Age (years) (m ± SD)	53.3 ± 13.4	48.1 ± 12.9	0.201
Risk factors, *n* (%)			
Hypertension	6 (30.0)	1 (4.2)	0.055
Diabetes	1 (5.0)	0	0.455
Current/previous smoking	3 (15.0)	2 (8.3)	0.828
Alcohol abuse	2 (10.0)	2 (8.3)	1.000
Onset symptoms, *n* (%)			
Pain	16 (80.0)	20 (83.3)	1.000
Paresthesia	12 (60.0)	15 (62.5)	0.865
Motor weakness	12 (60.0)	10 (41.7)	0.226
Sphincter dysfunction	0	3 (12.5)	0.300
Duration of symptoms(months) (IQR)	12.0 (3.5, 24.0)	8.0 (2.0, 48.0)	0.953
Preprocedural ASIA scale, *n* (%)			0.155
C	0	1 (4.2)	
D	13 (65.0)	9 (37.5)	
E	7 (35.0)	14 (58.3)	
Location, *n* (%)			0.742
Cervical	8 (40.0)	6 (25.0)	
Thoracic	5 (25.0)	7 (29.2)	
Thoracolumbar	2 (10.0)	2 (8.3)	
Lumbar	5 (25.0)	8 (33.3)	
Lumbar and lumbosacral	0	1 (4.2)	

EA, epidural approach; CA, conventional approach; SD, standard deviation; IQR, interquartile range.

Tumor distribution by spinal region was: cervical (31.8%, 14/44, including 4 were located at C1/2 level), lumbar (29.5%, 13/44), thoracic (27.3%, 12/44), thoracolumbar (9.1%, 4/44), and lumbosacral (2.3%, 1/44). According to Eden classification, 6.8% (3/44) were Type 1 and 93.2% (41/44) were Type 2 tumors. Detailed tumor characteristics are summarized in Table 1.

### 3.2 Procedure-related data

**Figure 2:**
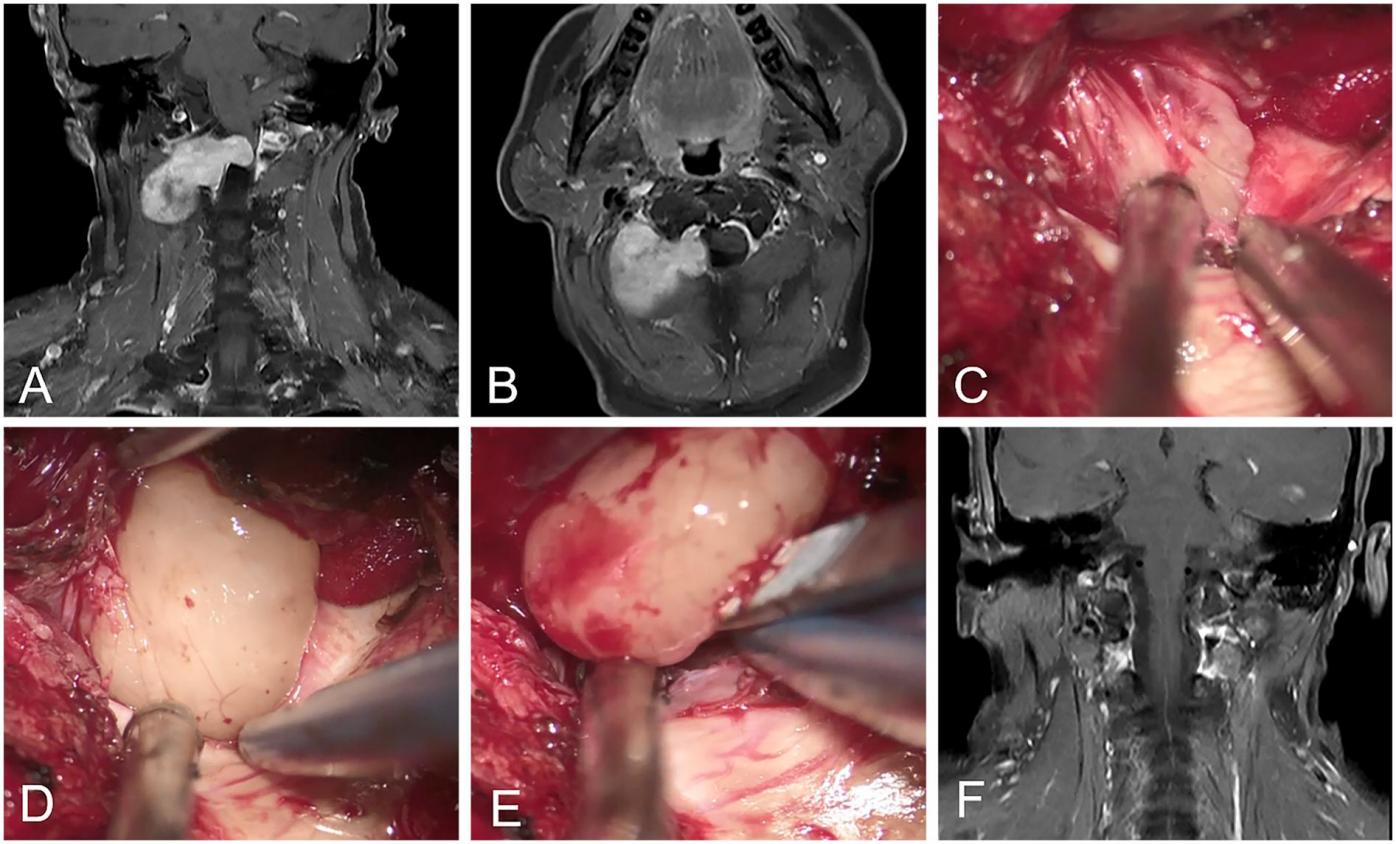
Representation of C1–C2 schwannomas. **(A,B)** The preprocedural MRI scan revealed the dumbbell-shaped schwannomas located in the right cervical spine at the C1–C2 intervertebral foramen. **(C,D)** The tumor was gently retracted laterally, and its interface with the dura mater was meticulously dissected. **(E)** The intradural tumor was successfully exteriorized via the trans-epidural approach. **(F)** Postprocedural MRI revealed no tumor residual.

All procedures were successfully completed. Postoperative MRI demonstrated gross total resection in 42 patients (95.5%), with two residual cases occurring in the conventional approach group. Among the 20 patients in the epidural approach group, transforaminal approach was used in 5 patients and the hemilaminectomy approach was used in 15 patients. The gross total resection rates were 100% (20/20) in the epidural approach group vs. 91.7% (22/24) in the conventional group (*P* = 0.493). Parent nerve preservation was achieved in 33 patients (75.0%), while intentional nerve root sacrifice was performed in 11 cases (25.0%) without causing new neurological deficits. A representative case is illustrated in Figure 2.

Internal fixation was required in 1 patient (5.0%) in the epidural approach group due to the massive, multi-level characteristics of the tumor, compared with 15 patients (62.5%) in the conventional group. The median blood loss was significantly lower in the epidural approach group (200 mL; IQR: 125–375) than in the conventional approach group (500 mL; IQR: 300–900) (*P* < 0.001). No patients required blood transfusion in the epidural approach group, whereas 10 patients (41.7%) in the conventional approach group received transfusions (*P* = 0.003). The median procedure time was 265 (IQR: 240–347) minutes in the epidural approach group, compared to 408 (IQR: 269–540) minutes in the conventional approach group. This difference was statistically significant (*P* = 0.009). Detailed procedural outcomes are presented in Table 2.

**Table 2:** Procedure related date, in-hospital serious adverse events and outcomes.

Variables	EA group (*n* = 20)	CA group (*n* = 24)	*P* value
Gross total resection, *n* (%)	19 (95.0)	24 (100.0)	0.455
Parent nerve sacrifice, *n* (%)	4 (20.0)	4 (16.7)	1.000
Intraprocedural estimated blood loss, (mL) (IQR)	200 (125, 375)	500 (300, 900)	<0.001
Blood transfusion, *n* (%)	0	10 (41.7)	0.003
In-hospital SAEs, *n* (%)	3 (15.0)	9 (37.5)	0.095
Severe pulmonary infection	2 (10.0)	2 (8.3)	1.000
Severe urinary tract infection	1 (5.0)	3 (12.5)	0.738
Deep vein thrombosis	0	3 (12.5)	0.300
CSF leakage	0	1 (4.2)	1.000
Neurological deterioration	0	2 (8.3)	0.493
CNS infections	0	4 (16.7)	0.165
ASIA scale upon discharge, *n* (%)			0.572
D	2 (10.0)	5 (20.8)	
E	18 (90.0)	19 (79.2)	
Efficacy at discharge, *n* (%)			0.151
Improvement	18 (90.0)	16 (66.7)	
Stable	2 (10.0)	6 (25.0)	
Neurological deterioration	0	2 (8.3)	
Follow-up duration, (months) (IQR)	29.0 (11.0, 46.0)	27.5 (8.0, 60.0)	0.878
ASIA scale at follow-up, *n* (%)			0.493
D	0	2 (8.3)	
E	20 (100.0)	22 (91.7)	

EA, epidural approach; CA, conventional approach; SD, standard deviation; IQR, interquartile range; SAEs, serious adverse events; CSF, cerebrospinal fluid leakage; CNS, central nervous system; ASIA, American spinal injury association.

### 3.3 In-hospital SAEs

Twelve patients (27.3%) experienced at least one serious adverse event (SAE) before discharge, comprising 3 cases (15.0%) in the epidural approach group and 9 cases (37.5%) in the conventional group. Although the conventional group showed a numerically higher SAE incidence, this difference did not reach statistical significance (*P* = 0.095; Table 3). The SAEs included: severe pulmonary infections (9.1%, 4/44), urinary tract infections (9.1%, 4/44), lower limb deep vein thrombosis (6.8%, 3/44), cerebrospinal fluid leakage (2.3%, 1/44), neurological deterioration (4.5%, 2/44), and CNS infections (9.1%, 4/44). Detailed SAE data are presented in Tables 2, 3.

**Table 3:** Serious adverse events details.

No.	Treatment strategy	SAE details	ASIA scale		
			Baseline	At discharge	At last FU
1	EA	Urinary tract infection	D	D	E
2	CA	Deep vein thrombosis	E	E	E
3	CA	Deep vein thrombosis; CNS infection	D	D	E
4	EA	Pulmonary infection	D	E	E
5	CA	CSF leakage	E	E	E
6	CA	Pulmonary infection; urinary tract infection	E	E	E
7	CA	Deep vein thrombosis	E	E	E
8	CA	Urinary tract infection; CNS infection; neurological deterioration	E	D	E
9	EA	Pulmonary infection	E	E	E
10	CA	CNS infection	E	E	E
11	CA	CNS infection; neurological deterioration	D	D	D
12	CA	Pulmonary infection; urinary tract infection	E	E	E

EA, epidural approach; CA, conventional approach; SAE, serious adverse event; CSF, cerebrospinal fluid leakage; FU, follow-up; ASIA, American spinal injury association; CNS, central nervous system.

Univariate analysis identified potential associations between SAEs and surgical approach (*P* = 0.095), intraprocedural blood transfusion (*P* = 0.025), and intraprocedural blood loss ≥350 mL (*P* = 0.009) (Table 4). Multivariate analysis demonstrated that intraprocedural blood loss ≥350 mL was independently associated with SAEs (OR 6.6, 95% CI 1.5–29.7; *P* = 0.014), while surgical approach (OR 1.5, 95% CI 0.2–9.4; *P* = 0.661) and intraprocedural blood transfusion (OR 3.0, 95% CI 0.5–19.6; *P* = 0.251) showed no significant association.

**Table 4:** Univariate analyses of factors influencing in-hospital serious adverse events.

Variables	No SAE group (*n* = 32)	SAE group (*n* = 12)	*P* value
Treatment strategy			0.095
EA	17 (53.1)	3 (25.0)	
CA	15 (46.9)	9 (75.0)	
Male, *n* (%)	19 (59.4)	8 (66.7)	0.924
Age (years) (m ± SD)	49.6 ± 12.7	52.9 ± 14.9	0.461
Risk factors, *n* (%)			
Hypertension	6 (18.8)	1 (8.3)	0.705
Diabetes	1 (3.1)	0	1.000
Current/previous smoking	3 (9.4)	2 (16.7)	0.884
Alcohol abuse	2 (6.3)	2 (16.7)	0.630
Onset symptoms, *n* (%)			
Pain	28 (87.5)	8 (66.7)	0.247
Paresthesia	18 (56.3)	9 (75.0)	0.430
Motor weakness	17 (53.1)	5 (41.7)	0.498
Sphincter dysfunction	2 (6.3)	1 (8.3)	1.000
Duration of symptoms(months) (IQR)	12.0 (2.5, 30.0)	10.5 (5.0, 84.0)	0.541
Preprocedural ASIA scale, *n* (%)			0.116
C	1 (3.1)	0	
D	18 (56.3)	4 (33.3)	
E	13 (40.6)	8 (66.7)	
Location, *n* (%)			0.496
Cervical	10 (31.3)	4 (33.3)	
Thoracic	7 (21.9)	5 (41.7)	
Thoracolumbar	4 (12.5)	0	
Lumbar	10 (31.3)	3 (25.0)	
Lumbar and lumbosacral	1 (3.1)	0	
Parent nerve sacrifice, *n* (%)	7 (21.9)	4 (33.3)	0.696
Intraprocedural estimated blood loss, (mL) (IQR)			0.009
˂350 ml	22 (68.8)	3 (25.0)	
≥350 ml	10 (31.3)	9 (75.0)	
Blood transfusion, *n* (%)	4 (12.5)	6 (50.0)	0.025

EA, epidural approach; CA, conventional approach; SD, standard deviation; IQR, interquartile range; SAE, serious adverse event; CSF, cerebrospinal fluid leakage; ASIA, American spinal injury association.

### 3.4 Outcomes

At discharge, 34 (34/44, 77.3%) patients reported improvement of their symptoms, while 8 (8/44, 18.2%) patients remained stable, and 2 (2/44, 4.5%) patients experienced neurological deterioration. All patients received clinical follow-up, with the median follow-up duration of 28.0 (9.5, 51.0) months. During the follow-up period, no new neurological or procedure-related symptoms occurred in any of the patients. Among the 2 patients with neurological deterioration, one returned to the preprocedural level, while the other improved. No spinal instability was detected throughout the follow-up period. Imaging follow-up with enhanced MRI was performed in 27 patients, with the median follow-up duration of 12 (9, 12) months. No tumor recurrence was detected. The detailed outcomes are summarized in Table 2.

## 4 Discussion

In the present study, the gross total resection rates were similar between the epidural approach group and the conventional approach group, demonstrating that both procedures exhibit comparable efficacy. The incidence of SAEs in the conventional approach group was numerically higher than that in the epidural approach group. The intraprocedural blood loss and the incidence of intraprocedural blood transfusions were significantly higher in the conventional approach group than those in the epidural approach group. Multivariate analysis revealed that intraprocedural estimated blood loss ≥ 350 mL was significantly associated with SAEs during hospitalization.

Regarding the origin of dumbbell-shaped tumors, for those involving both intradural and extradural compartments, the intradural component extends through the dural sleeve of the nerve root into the intervertebral foramen, forming a tumor that spans the dural space in a dumbbell configuration, and dumbbell tumors originating in the paravertebral segment of the spinal nerve usually do not involve the dural, but extend toward the intervertebral foramen (12). Study by Ozawa et al. (2) has shown that dumbbell-shaped tumors located in the cervical spine are more common than those in the thoracic and lumbar spine, accounting for about 44% of dumbbell-shaped tumors in all locations, which may be due to the fact that schwannoma, the most common type of dumbbell-shaped tumor, mostly occurs in the upper cervical nerve roots and less frequently originates from the thoracic and lumbar nerve roots. Dumbbell-shaped tumors involving both the intradural and extradural spaces are relatively common, accounting for approximately 40% of all types of dumbbell-shaped tumors (2). The conventional open surgery via posterior midline approach requires extensive dissection of paraspinal muscles, laminar exposure, and either hemilaminectomy or bilateral laminectomy combined with ipsilateral facetectomy to achieve adequate tumor exposure. Previous studies have shown that conventional open surgical treatment for dumbbell-shaped tumors achieves a gross total resection rate of approximately 70% (1, 15), with a postprocedural complication rate of about 3%–10% (1, 16). For patients with significant bone destruction, internal fixation is usually required to ensure the stability of the spine (2, 7, 15). However, previous studies have reported that approximately 10% of patients may develop spinal deformity postprocedurally (17). A study by Montano et al. (17) has shown that laminectomy may be associated with new-onset or worsening of spinal deformity after resection of intradural spinal tumors. Traditional highly invasive surgery, in many cases, may pose risks of surgery-related infections, blood loss, and spinal cord injury, compromise the stability of the spine, and lead to motor and sensory deficits (5–8, 11, 17). To simplify the surgical procedures, reduce surgical trauma, and minimize the damage of the surgery to the spinal cord and the osseous structures of the spine, the exploration of new treatment methods for spinal dumbbell-shaped tumors is particularly crucial.

For dumbbell-shaped tumors, due to their unique imaging characteristics and local anatomical structures, the intraspinal canal portion of the dumbbell-shaped tumor can be resected through the intervertebral foramen or hemilaminectomy without the need for bilateral laminectomy. To a certain extent, it could reduce the damage to the paravertebral tissues and promote the recovery after the surgery. Zhang et al. (12) have retrospectively enrolled a case series of 34 patients with cervical extra-intraspinal neuromas who had been treated with the enlarged intervertebral foramen. All patients retained the osseous structures of the affected intervertebral foramina, thereby eliminating the need for additional internal fixation or bone grafting; moreover, gross total resection was achieved in 91.2% of cases, with no recurrence observed during contrast-enhanced MRI follow-up among these patients. In addition, they have also reported that 11.8% of the patients experienced adverse events after the surgery, and 5.9% of the patients had postprocedural adverse neurological events. Maragkos et al. (4) described a novel treatment approach. To minimize the disruption of spinal stability caused by the surgery, they carried out a small hemilaminectomy, along with a minimal medial facetectomy, while taking great care to preserve the pars interarticularis. The results showed that the tumors within the spinal canal of all patients were completely resected. However, 16.7% of the patients had residual tumors outside the distal intervertebral foramen. To further simplify the surgical procedures and reduce surgical trauma, we have proposed an epidural trans-dural sleeve approach without dural incision for the resection of intra-extradural spinal dumbbell-shaped tumors. The intradural portions of the tumor were gently retracted outward, and this technique avoids laminectomy and thereby preserves spinal stability. Additionally, it simplifies the surgical procedure while reducing the risks associated with dural opening, such as spinal cord injury, intraprocedural bleeding, and CSF leakage.

Our study demonstrated that the incidence of in-hospital SAEs was numerically higher in the conventional approach group compared to the epidural approach group (37.5% vs. 15.0%), but the difference did not reach statistical significance. However, the intraprocedural blood loss and the intraprocedural estimated blood loss were significantly higher in the conventional approach group, and multivariate analysis showed that intraprocedural estimated blood loss ≥ 350 mL was significantly associated with SAEs during hospitalization. Previous studies have shown that excessive intraprocedural blood loss and blood transfusion were significantly associated with increased morbidity and mortality in patients undergoing spinal surgery (18). Although blood transfusion is relatively safe when essential, it may still be associated with adverse events, including transfusion reactions, transfusion-related acute lung injury, postprocedural complications, and infections (18, 19). Moreover, the study by Purvis et al. (20) found that in spinal surgery, the percentage change in hemoglobin levels from preoperative values to the lowest postoperative levels was significantly associated with the risk of periprocedural complications and hospital-acquired infections (19). A decrease in hemoglobin may have certain negative impacts on highly oxygen-consuming organs such as the heart, brain, and kidneys. These patients typically experience greater surgical trauma and prolonged postprocedural bed rest, which often contributes to an elevated risk of surgical site infections (19).

Postprocedural CSF leakage is one of the major complications for intraspinal canal tumors, which often leads to poor outcomes. Moreover, compared with non-dumbbell-shaped tumors, dumbbell-shaped tumors are associated with a higher incidence of CSF leakage (4, 21–24). The incidence of CSF leakage is related to the size of the tumor, which is caused by the dural defect after the resection of the intradural-extradural tumor. Previous studies have reported that the incidence of CSF leakage after the procedure for spinal dumbbell-shaped tumors is approximately 7.7%–40.1% (7, 13, 24). Yuan et al. (21) reported a case of cerebrospinal fluid leakage following the resection of a cervical spinal canal dumbbell-shaped schwannoma. The patient subsequently developed CNS infection, hemorrhagic cerebral infarction, unilateral limb sensorimotor dysfunction, and language impairment, resulting in a poor outcome. This case highlights the need for vigilance against cerebrospinal fluid leakage during surgical resection of dumbbell-shaped tumors. Ito et al. (24) proposed a novel dural suturing method following laminotomy for the resection of intraspinal dumbbell-shaped tumors. Initial results demonstrated certain therapeutic efficacy, but this surgical approach required laminotomy and caused significant damage to spinal stability. In this study, during the resection of intradural tumors, the tumors were extracted by outward traction along their natural pathways without requiring additional dural incision. This approach minimized damage to the dura mater and internal arachnoid membrane structures, thereby reducing the incidence of postprocedural CSF leakage. Notably, no cases of postprocedural CSF leakage occurred in the epidural approach group.

For schwannomas, the optimal treatment standard is complete tumor resection while preserving the nerve root and neurological function. However, in patients with dumbbell-shaped tumors, preserving the nerve root requires expanding the tumor exposure, increasing trauma, and raising the probability of tumor subtotal resection. There is still no conclusive conclusion in the research on whether sacrificing the nerve root during the resection of dumbbell-shaped schwannoma will pose risks in terms of permanent motor dysfunction and neuropathic pain for patients (13, 23). Previous studies have shown that sacrificing the parent nerve root during resection of spinal dumbbell tumors did not increase the incidence of postprocedural neurological deficits in patients (13, 25). This may be related to the gradual loss of function of the parent nerve root, and the cross-innervation of adjacent nerve roots may also have a compensatory effect on it. In our study, 11 of 44 patients with schwannomas experienced the parent nerve root sacrifice, and no permanent motor dysfunction or neuropathic pain was noted. It is suggested that for spinal dumbbell-shaped tumors, sacrificing the involved nerve roots to simplify the surgical procedure and improve the gross total resection rate of the tumor is acceptable.

The main limitations of this study include its retrospective design, relatively small sample size, non-randomized nature, and inherent selection bias. Although our results demonstrated a numerically lower incidence of SAEs in the epidural approach group compared to conventional approach group, the difference did not reach statistical significance. Furthermore, the limited sample size may reduce the reliability of multivariate logistic regression analyses, and the inherent potential biases of retrospective studies remain unavoidable. Thus, a prospective comparative trial with a large sample size, or developing a standardized surgical selection guideline based on MRI features would be of significant clinical value.

## 5 Conclusion

The epidural trans-dural sleeve approach may be a feasible option for treating intra-extradural spinal dumbbell-shaped tumors, demonstrating similar gross total resection rates while offering relatively lower intraprocedural blood loss and transfusion rates compared to conventional approaches. There was a potential trend toward lower SAE rates in the epidural approach group compared to conventional laminectomy and dural incision. Intraprocedural estimated blood loss ≥ 350 mL was significantly associated with SAEs during hospitalization.

## Data Availability

The original contributions presented in the study are included in the article/Supplementary Material, further inquiries can be directed to the corresponding author.
